# Detection and molecular typing of *Leishmania tropica* from *Phlebotomus sergenti* and lesions of cutaneous leishmaniasis in an emerging focus of Morocco

**DOI:** 10.1186/1756-3305-6-217

**Published:** 2013-07-26

**Authors:** Malika Ajaoud, Nargys Es-sette, Salsabil Hamdi, Abderahmane Laamrani El-Idrissi, Myriam Riyad, Meryem Lemrani

**Affiliations:** 1 Laboratoire de Parasitologie et Maladies Vectorielles, Institut Pasteur du Maroc, 1 Place Louis Pasteur, 20360 Casablanca, Morocco; 2 CEDoc des Sciences de la Santé, Faculté de Médecine et de Pharmacie, Université Hassan II, Casablanca, Morocco; 3 Service des Maladies Parasitaires, Direction de l’Epidémiologie et de la Lutte contre les Maladies, Ministère de la Santé, Rabat, Morocco; 4 Laboratoire de Parasitologie-Mycologie, Faculté de Médecine et de Pharmacie, Université Hassan II, Casablanca, Morocco

**Keywords:** Cutaneous leishmaniasis, *Phlebotomus sergenti*, Genotyping, *Leishmania tropica*, Morocco

## Abstract

**Background:**

Cutaneous leishmaniasis is an infectious disease caused by flagellate protozoa of the genus *Leishmania*. In Morocco, anthroponotic cutaneous leishmaniasis due to *Leishmania tropica* is considered as a public health problem, but its epidemiology has not been fully elucidated. The main objective of this study was to detect *Leishmania* infection in the vector, *Phlebotomus sergenti* and in human skin samples, in the El Hanchane locality, an emerging focus of cutaneous leishmaniasis in central Morocco.

**Methods:**

A total of 643 sand flies were collected using CDC miniature light traps and identified morphologically. *Leishmania* species were characterized by ITS1 PCR-RFLP and ITS1-5.8S rRNA gene nested-PCR of samples from 123 females of *Phlebotomus sergenti* and 7 cutaneous leishmaniasis patients.

**Results:**

The sand flies collected consisted of 9 species, 7 of which belonged to the genus *Phlebotomus* and two to the genus *Sergentomyia*. *Phlebotomus sergenti* was the most predominant (76.67%).

By ITS1 PCR-RFLP *Leishmania tropica* was found in three *Phlebotomus sergenti* females and four patients (4/7). Using nested PCR *Leishmania tropica* was identified in the same three *Phlebotomus sergenti* females and all the 7 patients. The sequencing of the nested PCR products recognized 7 haplotypes, of which 6 have never been described.

**Conclusions:**

This is the first molecular detection and identification of *Leishmania tropica* in human skin samples and *Phlebotomus sergenti* in support of its vector status in El Hanchane. The finding of seven *Leishmania tropica* haplotypes underscores heterogeneity of this species at a high level in Morocco.

## Background

In Morocco, *Leishmania major*, *Leishmania tropica* and less frequently *Leishmania infantum* cause cutaneous leishmaniasis (CL). Zoonotic CL caused by *L. major* zymodeme MON-25 has been known since 1914 and is largely confined to the arid pre-Saharan regions [1]. In the last decades, outbreaks of CL have been reported repeatedly due to reactivation of old southern foci, amounting to several thousand cases [2]. Amongst the 3 clinically important *Leishmania* species, *L. tropica* has the largest geographic distribution in Morocco and is considered as a public health threat by the Ministry of Health. This anthroponotic form of CL was reported for the first time in 1989 [3]. There after hypoendemic rural foci were identified scattered around in the sub-arid area of central Morocco, caused by a great diversity of this species [4,5]. Recently, CL caused by *L. tropica* emerged in several new foci in Northern Morocco. Indeed, in 1995, Taza province registered a rapid expansion of CL due to *L. tropica* MON-102 [6,7]. In 2001, about 1600 people were also infected with *L.tropica* in Fes province [8]. Molecular examination of samples from skin lesions collected from different regions has shown that *L. tropica* is present in a large part of the kingdom, and for the first time even in regions previously known only as *L. major* CL foci [8].

The epidemiology of leishmaniasis caused by *L.tropica* in Morocco has not been fully elucidated. The disease is often described as being anthroponotic and most CL cases occur in and around densely populated cities [6,8]. However, the low frequency of the disease in semi rural locations and the sudden occurrence of small outbreaks suggest that the disease may be zoonotic in some cases [6]. The rapid spread of the parasite, with the increasing number of cases begs epidemiological investigations.

Molecular methods are increasingly employed for diagnostic and epidemiological purposes in order to confirm *Leishmania* infection and to characterize the parasites at the species or genotype level in hosts and vectors. The detection of *Leishmania* parasites by PCR methods is highly specific and sensitive, with values reaching up to 100% [9]. Several techniques have been described, such as PCR-restriction fragment length polymorphism (RFLP), sequence analysis of multicopy genes and intergenic spacer regions (ITS), DNA fingerprinting and randomly amplified polymorphic DNA (RAPD) [10] and multilocus sequencing typing (MLST), which is a powerful technique for phylogenetic studies in *Leishmania*[11] and may substitute isoenzyme analysis. Accurate and sensitive diagnostic and identification procedures are required to distinguish *Leishmania* species/strains whose geographic distribution can overlap, which is crucial for adequate treatment and appropriate public health control measures. Based on a literature search, we selected two PCR methods shown to have sensitivity and specificity for detecting *Leishmania*: ITS1 PCR-RFLP and the nested PCR of ITS rDNA genes. Both methods were used to detect and identify the *Leishmania* parasite responsible for the recent cases of CL and their putative vector species in El Hanchane district, an emerging focus of CL in central Morocco.

## Methods

### Study area and sample collection

**Figure 1:**
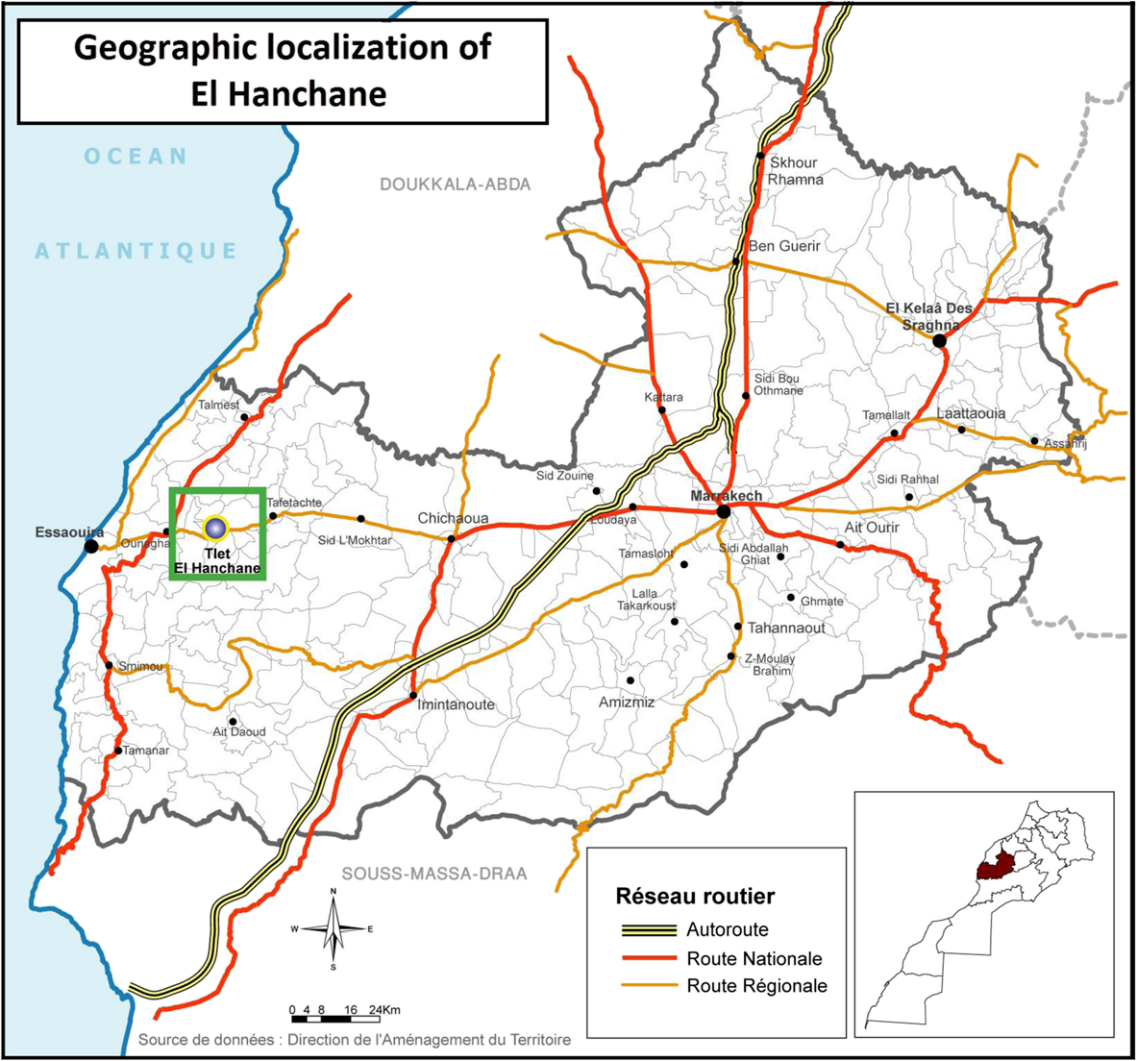
Map of Morocco showing El Hanchane locality: the study area.

In this study, sand flies were caught in El Hanchane, an emerging focus of CL according to the local health authorities. Since the first case reported in 2006, there has been an average of12 CL cases per year up to 2010. El Hanchane is a semi-rural locality, situated at an altitude of 290–300 m above sea level (31°31’11” N, 9°26’02” W) (Figure 1).

Sandflies were collected using CDC miniature light traps in domestic animal shelters and inside houses during 3 consecutive nights from July to September, 2011. The traps were placed approximately 1.5 meters above ground and set in the late afternoon for collection the following morning. The sand flies were then placed in 1.5 ml Eppendorf tubes and kept frozen at −80°C.

Tissue samples were taken from the edge of the lesions in 7 suspected CL patients at the health center in El Hanchane during 2011. Lesion smears were fixed with absolute methanol, and then stained with Giemsa for CL diagnosis. The whole slides were analyzed twice with a 100X immersion objective.

Microscopically confirmed CL patients received free intra lesional injections of meglumine antimoniate (Glucantime®; Sanofi -Aventis, Bridgewater, NJ, USA) according to the protocol of the Moroccan Ministry of Health [2].

Informed written consent was obtained from patients or from parents if the patient was a minor. Approval for the study was provided by the Ethical Committee of Institut Pasteur of Morocco.

### Sand fly identification

Sand fly specimens were washed in sterile distilled water, the head and genitalia of individual sand flies were removed, and the remainder of the body was stored in sterile Eppendorf microtubes for DNA extraction. Specimens were mounted on microscope slides with the solution of Marc-André (30 g of chloral hydrate, 30 mL glacial acetic acid and 30 mL of distilled water) and identified using the taxonomic keys of Moroccan sand flies, particularly those of Boussaa [12].

### Extraction of DNA from sand flies and stained smears of human CL lesions

The total DNAs were isolated from the positive stained smears of 7 CL lesions and from 123 unengorged female *P. sergenti* specimens using the phenol-chloroform method. Then DNA samples were purified using the Qiagen kit (QIAquick PCR Purification Kit, Germany), according to the manufacturer’s instructions and quantitatively determined using NanoDrop (Thermo Scientific) before dilution to a final concentration of 50 ng/μL.

### ITS1 PCR-RFLP of *Leishmania* species

The DNA samples from *P. sergenti* females and CL patient lesions were examined for the *Leishmania*-specific ribosomal internal transcribed spacer 1 region (ITS1) by PCR amplification using the primer pair L5.8S and LITSR followed by RFLP (restriction fragment length polymorphism) analysis as described by Schonian *et al.*[13].

The cycling conditions were 94°C for 2 min followed by 32 amplification cycles, each consisting of three steps: denaturation at 94°C for 20 s, annealing at 53°C for 30 s and extension at 72°C for 1 min, followed by a final extension at 72°C for 6 min in the thermocycler (S1000™ Thermal Cycler, Bio-Rad).

PCR products were digested with the restriction endonuclease *Hae*III for 2 h at 37°C. Restriction fragments were separated by electrophoresis on a 2% agarose gel and compared with those of WHO reference strains of *L. major* (MHOM/SU/73/5ASKH), *L. tropica* (MHOM/SU/74/K27) and *L. infantum* (MHOM/TN/80/IPT1).

### Nested PCR for amplifying ITS1-5.8S rDNA gene of *Leishmania* species

All female *P. sergenti* and CL patient lesions were further screened for infection with *Leishmania* species by nested PCR of the DNA samples for the presence of its ITS-rDNA gene. A two-stage PCR was carried out in two separate tubes [14,15]. The first-stage PCR used the forward primer IR1 with the reverse primer IR2 [16], and the second-stage PCR used the nested forward primer ITS1F with the nested reverse primer ITS2R4 [15].

The first amplification reaction in a total volume of 20 μL, contained 1X PCR Rxn Buffer (Invitrogen), 1.5 mM MgCl_2_, 60 μM of each dNTP, 1 μM primer IR1, 1 μM primer IR2, 1 unit Taq DNA polymerase (Invitrogen), and 50 ng template DNA. The mixture was incubated in thermocycler (S1000™ Thermal Cycler, Bio-Rad) under the following conditions: an initial denaturation at 94°C for 3 min, followed by 37 cycles each consisting of three steps: 30 s at 94°C (denaturation), 30 s at 58°C (annealing) and 90 s at 72°C (extension). After the last cycle, the extension step was continued for a further 10 min.

The products obtained were subsequently subjected to nested PCR amplification as follows: the reaction mixtures of 20 μL each contained the same reagents as the first-stage PCR, except that the primers were now primer ITS1F and primer ITS2R4, and the target DNA was 1 μL of the first-stage PCR reaction products. The reaction was programmed, as described for the first-stage PCR. Cross-contamination was monitored by negative controls for sample DNAs and solutions of all PCR reagents used.

The final PCR products of 400 bp were purified using the Exonuclease I/Shrimp Alkaline Phosphatase (GE Healthcare, US) before sequencing by using BigDye Terminator version 3.1 Cycle Sequencing kit (Applied Biosystems, Foster City, CA, USA) and an ABI PRISM 3130 DNA automated sequencer (Applied Biosystems). Sequencing data were analyzed using SeqScape v.2.5 software (Applied Biosystems). Sequences were aligned using the multiple alignment program MEGA5 and GENtle v.1.9.4 software. A phylogenetic tree was constructed by using the Neighbor-Joining method in agreement with Kimura 2-parameter model with uniform rates for transitions and transversions. Bootstrap replicates were performed to estimate the node reliability, and values were obtained from 1,000 randomly selected samples of the aligned sequence data. Sequences were compared with entries retrieved from Genbank.

## Results

### Morphological identification of sand flies

A total of 643 sand flies were trapped using CDC miniature light traps. All sand flies were morphologically identified using a standard key of Moroccan sand flies modified by Boussaa [12]. Nine species, including seven in the genus *Phlebotomus* and two in the genus *Sergentomyia* were identified. *P.sergenti* was the most prevalent species with 493 specimens (76.67%) (273 females/220 males), followed by *P*. *longicuspis* (11.51%) (Table 1).

**Table 1:** Diversity and relative abundance of sand flies species collected in El Hanchane locality

**Species**	**Female**	**Male**	**Total**	**(%)**
*P. sergenti*	273	220	493	76.67
*P. longicuspis*	52	22	74	11.51
*P. alexandri*	22	0	22	3.42
*P. perniciosus*	4	4	8	1.24
*P. kazeruni*	2	0	2	0.31
*P. langeroni*	0	4	4	0.62
*P. bergeroti*	1	0	1	0.16
*S. minuta*	19	6	25	3.89
*S. antennata*	1	13	14	2.18
**Total**	**374**	**269**	**643**	**100%**

#### *Leishmania* genotyping

**Figure 2:**
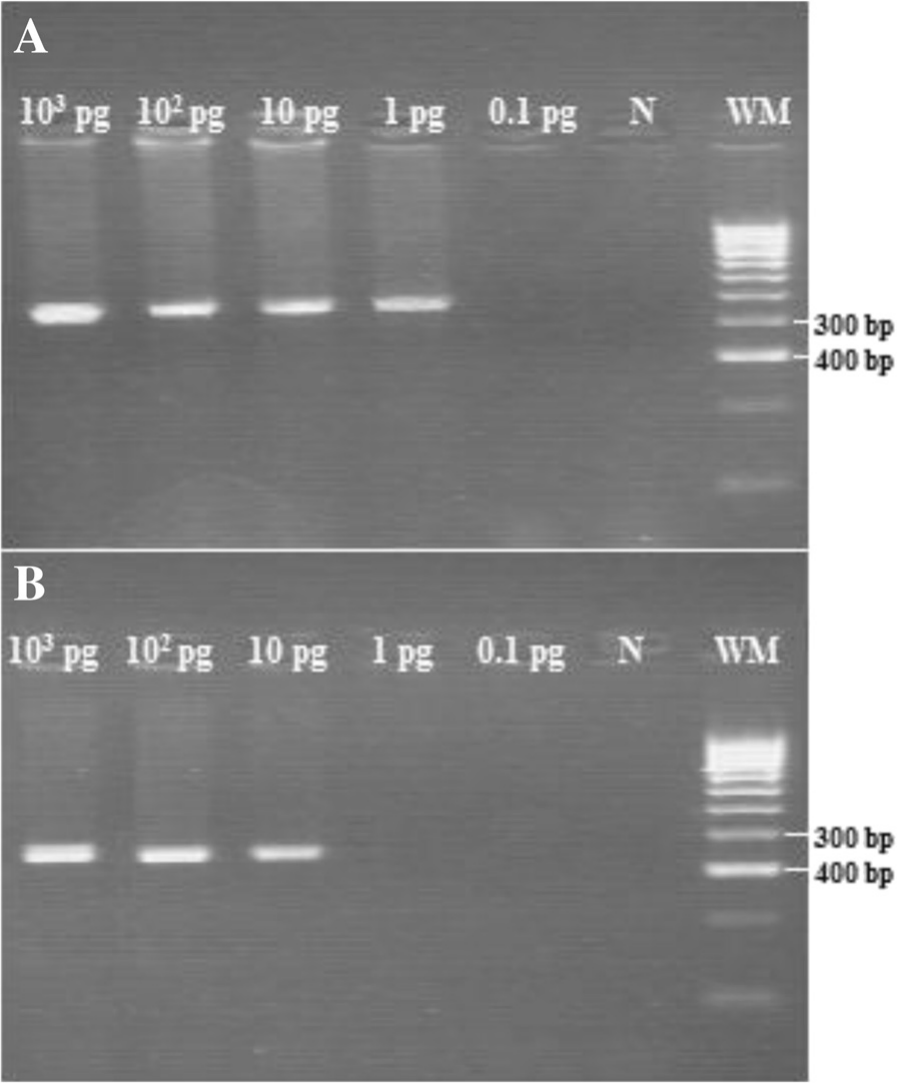
**Sensitivity analysis of nested PCR and ITS1-PCR.***L. tropica* DNA was diluted 10-fold starting from 10^3^ pg and tested for amplification. The *L. tropica* nested PCR amplify the 400 bp product **(A)**, and ITS1-PCR amplify about 320 bp product **(B)**. N: Negative control. 100 bp weight molecular (WM) (Bioline).

The first step of this genotyping was to identify the *Leishmania* species from sandflies and humans: for this purpose we used the ITS1 PCR-RFLP. The second step by nested PCR will allow enhancing the sensitivity of our diagnosis. Indeed we demonstrated that the nested PCR allowed the detection of 1 pg/μL DNA, whereas the ITS1-PCR detected 10 pg/μL DNA extracted from *L.tropica* reference strain (MHOM/SU/74/K27) (Figure 2).

#### *Leishmania* species identification by ITS1 PCR-RFLP

Three out of 123 female *P. sergenti* tested for *Leishmania* infection were positive using the ITS1-PCR, the positive specimens produced a band of 320-bp. The digestion of the PCR product with endonuclease *Hae*III revealed the profiles of *L. tropica* (185 and 57/53 bp) for the 3 specimens.

Using ITS1-PCR followed by the digestion of amplicons, *Leishmania* parasites were successfully identified for 4 of the 7 positive stained slides of human CL lesions, the RFLP profiles are identical for the 4 samples, consisting of two bands (185 and 57/53 bp), identical to that of the *L. tropica* (MHOM/SU/74/K27).

#### *Leishmania* infections of *P. sergenti* and patients identified by nested PCR of ITS1-5.8S rDNA gene

**Figure 3:**
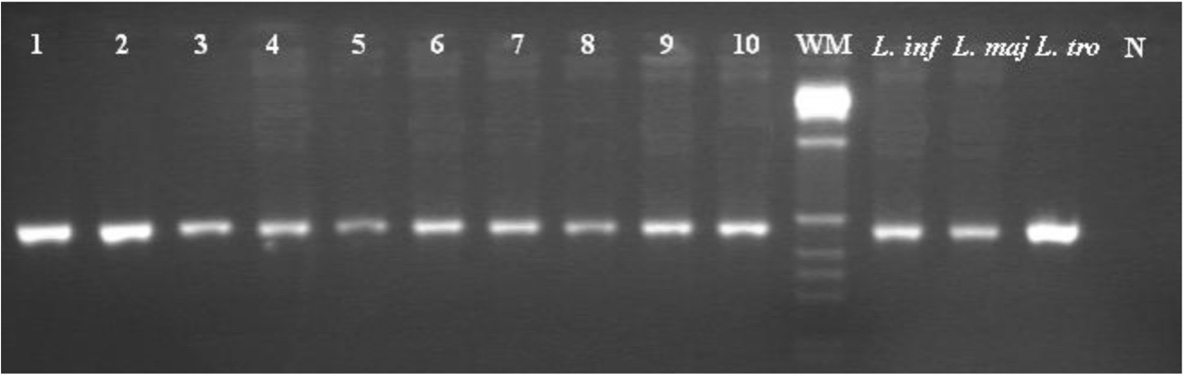
**Nested-PCR of ITS1-5.8S rDNA gene of *Leishmania *DNA from human samples and *P. sergenti *sand fly.** Lines 1–7: human samples; line 8–10: *P. sergenti* females. Reference strains: *L. infantum* (MHOM/TN/80/IPT1), *L. major* (MHOM/SU/73/5ASKH) and *L. tropica* (MHOM/SU/74/K27); N: Negative control; 1 kb weight molecular (WM) (Invitrogen).

Only the three female *P. sergenti* positive for *Leishmania* by ITS1-PCR were also found positive by the nested PCR of ITS rDNA gene, showing a band of approximately 400 bp; nested PCR of the seven positive CL patients examined for ITS-rDNA gene all produced a single band of 400 bp (Figure 3).

#### Sequencing and phylogenetic analysis of haplotype sequences of *Leishmania* ITS-rDNA

**Figure 4:**
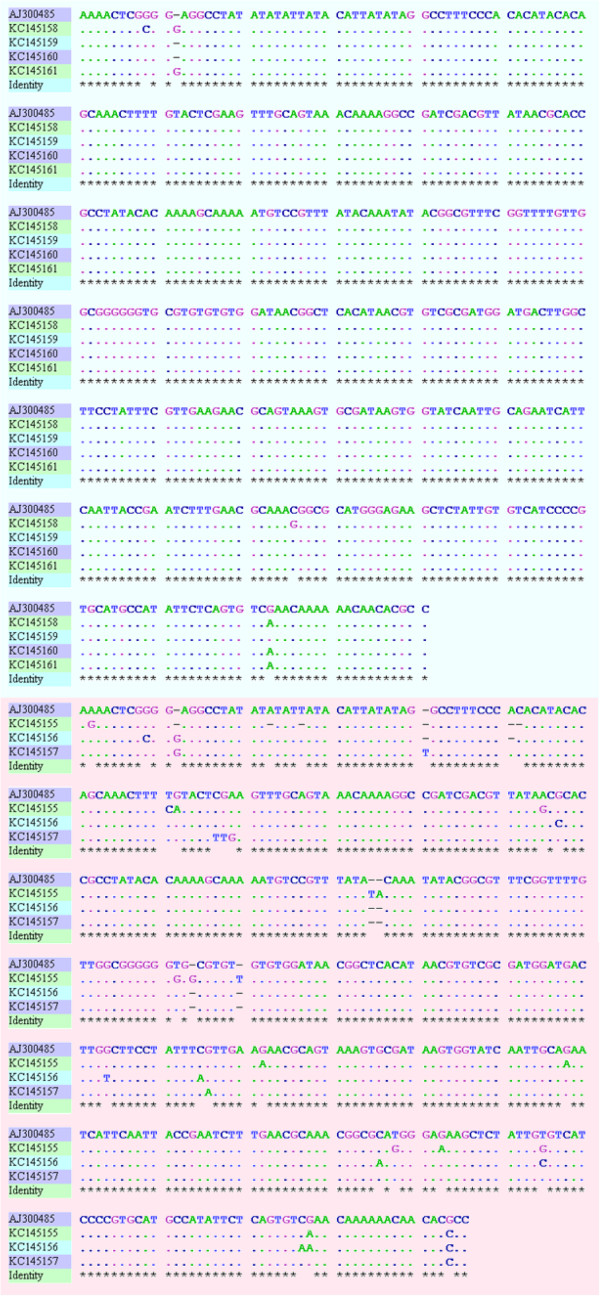
**ITS1-5.8S rDNA gene sequences alignment from patients and from *P. sergenti *with Tunisian haplotype.** Using GENtle software (v.1.9.4). Identities are denoted by points. Gaps are denoted by dashes.

**Figure 5:**
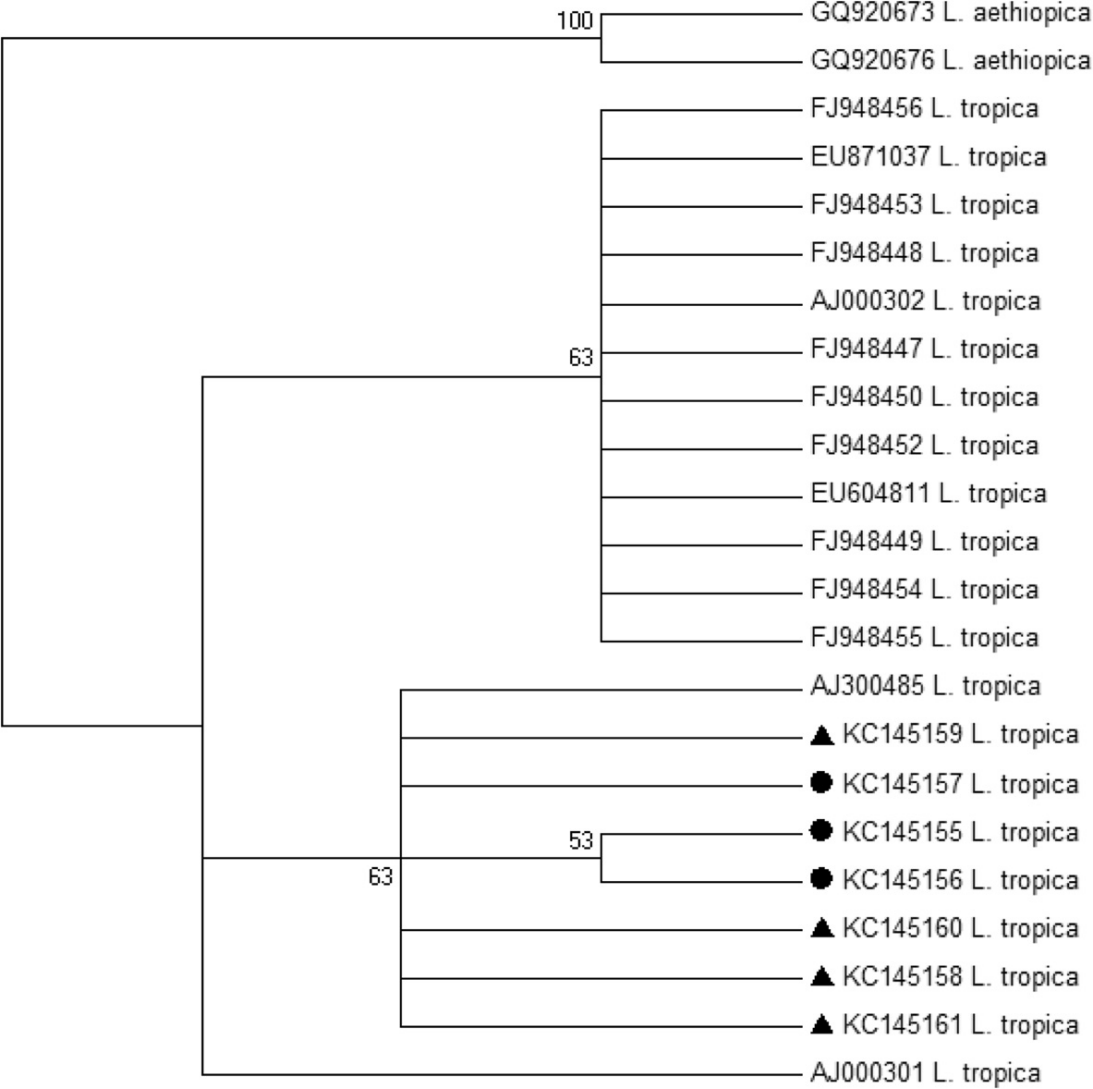
**Neighbor-Joining tree showing the relationships of the haplotypes of the ITS1-5.8S rDNA gene sequences of *Leishmania tropica *infecting *Phlebotomus sergenti *and patients, using MEGA 5 software, and it relates ITS1-5.8S rDNA gene haplotypes in GenBank.** The tree shown is based on the Kimura 2-parameter model of nucleotide substitution. Bootstrap values are based on 1,000 replicates. *L. aethiopica* was used as outgroup. Black triangles indicate haplotypes of *L. tropica* from patients and black disks haplotypes of *L. tropica* from *P. sergenti*.

Analysis of the ITS1-5.8S rDNA gene sequences obtained for 3 sand flies and 7 patients further confirmed that they all contain *L. tropica*. The sequences analyzed revealed 217 bp of ITS1 gene and 169 bp of 5.8S gene followed by 15 bp of ITS2. Alignment of these sequences revealed their sequence heterogeneity (Figure 4). The phylogenetic analysis of these sequences segregated seven *L. tropica* haplotypes: 3 from female *P. sergenti* and 4 from patients (Figure 5).

The haplotype KC145159 is identical to the Tunisian strain AJ300485, the haplotype KC145160, predominant in our CL patients (4/7), differs in one character, while KC145158 and KC145161 differ in two or four nucleotide positions, respectively.

*L. tropica* haplotypes KC145155; KC145156 and KC145157 from *P. sergenti* showed 95 to 98% similarity to the Tunisian haplotype AJ300485 showing 20, 10 and 7 base substitutions, respectively (Figure 4).

## Discussion

The main objectives of this study were to identify the sand fly vector and *Leishmania* species responsible for the recent outbreaks of CL in El Hanchane locality, a semi rural area in central Morocco where the first cases of CL appeared in 2006. Two molecular techniques were used for this purpose, ITS1 PCR-RFLP and nested PCR. Of the 7 samples of CL patients, 4 were successfully amplified and characterized by PCR-RFLP. The three remaining specimens were possibly either inhibited, or failed to amplify because of the low amount of DNA in stained slides. Several studies have exploited the amplification of the ITS-1 region for identifying *Leishmania* spp. in bone marrow aspirates or stained smears [13,17-19]. The major advantage of the ITS1-PCR is that species identification can be achieved by simple digestion of the PCR products. Thus, all clinically important species can be distinguished by their RFLP patterns. However, previous reports have shown that ITS1 PCR-RFLP is not sensitive enough for diagnostic purposes [20,21]. Using nested PCR, the 7 patients were all positive, confirming the better sensitivity of this technique [22].

In this survey, a total of 643 sand flies were collected indoors from July to September 2011 in an emerging focus of CL. The sandfly populations were mainly represented by *P. sergenti* (76.67%) and *P. longicuspis* (11.51%). Three unengorged female *P. sergenti* were infected by *Leishmania*, similar to *L. tropica* as demonstrated first by their ITS1PCR-RFLP profiles and then confirmed by the sequence identity of ITS-rDNA gene. In Morocco, *L. tropica* was first isolated from *P. sergenti* over three decades ago [4]. Since then and despite the large number of foci of CL due to *L. tropica* in Morocco, the vectors have never been identified with certainty because most epidemiological studies have focused on the identification of the parasite isolated from humans. *P.sergenti* is suggested as a vector based only on the circumstantial evidence of its ecology and its high frequency in an endemic area.

*Leishmania* infection of sand flies has classically been examined by dissecting freshly caught individual sand flies. This method allows the culture of *Leishmania* strains found in the sand fly gut; but it is time-consuming, needs dissecting expertise and a large number of specimens, since the *Leishmania* infection rate in sand flies is usually very low even in the highly endemic areas [23]. In recent years, molecular techniques have been used increasingly to identify *Leishmania* infection both in experimentally infected and field captured phlebotomine sand flies, as a sensitive and effective tool useful in epidemiological studies to facilitate strategic planning for the control of human leishmaniasis [24-26]. The increasing application of molecular techniques in this field has considerably reduced the time needed to obtain results; but such methods are underused in Morocco, despite the existence of CL foci in large areas and the lack of information for its vectors. This is the first report that *L. tropica* DNA was found in naturally infected *P. sergenti* from a CL focus of this country. The high density of *P.sergenti* indoors (i.e., anthropophilic) and their infection with *L. tropica* among unengorged females, suggest that this species plays a major role as the principal, if not the only vector in this locality.

*P. sergenti* is the confirmed vector of *L. tropica* throughout North Africa, Middle East and Central Asia [27-31]. However, *P. chabaudi* and the closely related species *P. riouxi* were also suspected as vectors based on their abundance in Tunisian and Algerian foci of CL due to *L. tropica*[32,33]. In Israel, in addition to *P. sergenti*, *P. arabicus* has been involved in the transmission of *L. tropica*[34,35]. None of these sand fly species were found in the present CL focus, where *P. sergenti* represents 76.67% of the total number of sand flies collected. On the other hand, the screening for *Leishmania* infection of the other sand fly species, separated into 5 monospecific pools, showed no PCR positives (data no shown), suggesting that *P. sergenti* is the only vector in this locality.

*L. tropica* is classically considered to be anthroponotic, however, in the present emerging focus, an anthroponotic transmission is very improbable, since the number of CL cases is too small, and their geographical distribution is too sparse and sporadic to consider humans as an adequate reservoir. Zoonotic outbreaks have been reported in many countries. Rock hyraxes were incriminated as reservoir hosts of *L. tropica* in Kenya and Namibia [36,37], in Israel *L. tropica* was isolated from rock hyrax (*Procavia capensis*): the strains were identical to those obtained from humans and sand flies in the same focus [38,39]. In southeast Tunisia gundi (*Ctenodactylus gundi*) was supported as a reservoir host of *L. tropica*[40,41], whereas in Algeria human CL caused by *L. killicki* is considered as a zoonotic disease with *P. sergenti* sand flies acting as vectors and gundi rodents (*Massoutiera mzabi*) as reservoirs [30]. In Morocco *L. tropica* has also been isolated from dogs in Azilal, a southern CL focus [42], but the reported infections in dogs were strictly cutaneous, the evolution duration of lesions was too short to consider dogs as the reservoir of the parasite.

*L. tropica* is recognized as a very heterogeneous species of *Leishmania* and intraspecific heterogeneity has been readily demonstrated by many investigators [43-45]. Microsatellite analysis revealed that two genetically very distinct populations of *L. tropica* co-exist within the same focus in Morocco: population ‘Morocco A’ is related to population ‘Asia’, whereas population ‘Morocco B’ is genetically closer to the other African populations [46].

In terms of isoenzyme profile variability, Morocco is characterized by the largest number of zymodemes ever described in *L. tropica*, with 8 zymodemes detected from human CL, dogs and the sandflies, e.g. *P. sergenti*[4,5,47]. In the present study, the DNA sequences of *L. tropica* isolated from *P. sergenti* showed a great heterogeneity, that was also observed in a southern Moroccan focus where *L.tropica* isolates from *P. sergenti* showed a wider range of zymodemes than isolates collected from the vertebrate hosts (dogs and humans) in the same region [42]: 74 *L. tropica* strains isolated from *P. sergenti* females were typed and corresponded to four zymodemes (MON-102: one strain; MON-107: 56 strains, MON-122: two strains; and MON-123: 15 strains). Only the first two zymodemes were also identified in humans and their frequencies were different in humans, dogs and the vector [4]. The zymodeme heterogeneity of strains isolated from vectors was also described with *L. infantum* isolates from *P. perniciosus* compared to vertebrate hosts in the same Spanish region [48]. Our results reinforce the observation that *L. tropica* is polymorphic; especially among those detected in *P. sergenti* compared to human samples.

## Conclusion

In conclusion, the ITS1 PCR-RFLP and the ITS1-5.8S rRNA gene nested PCR enable us to detect and to identify *L. tropica* in clinical samples and within sand flies. We have demonstrated that *L. tropica* is the causative agent of CL in this emerging focus of CL and that *P. sergenti* is the putative, but apparent vector of this species. The sequencing of the ITS1-5.8S rRNA gene amplicons allowed the identification of 6 new previously unknown sequence haplotypes, which under scores specific genetic heterogeneity of this species in Morocco.

## Competing interest

Authors declare that they have no competing interests.

## Authors’ contributions

MA: collected sandflies carried out the laboratory work of entomological and molecular studies, analyzed data, and prepared the manuscript. NE: contributed to the entomological studies and data analysis. SH: was involved in the collection of samples. AAE: is involved in the study design. MR: was involved in the review of the manuscript. ML: designed the study, contributed to interpretation, analyzed and finalized the manuscript. All the authors read and approved the final version of the manuscript.
